# Estimating shadow prices in economies with multiple market failures

**DOI:** 10.1371/journal.pone.0293931

**Published:** 2023-11-06

**Authors:** Alan Hernández-Solano, Véronique Sophie Avila-Foucat, George A. Dyer

**Affiliations:** 1 Instituto de Investigaciones para el Desarrollo con Equidad, Universidad Iberoamericana, Mexico City, Mexico; 2 Instituto de Investigaciones Económicas, Universidad Nacional Autónoma de México, Mexico City, Mexico; 3 El Colegio de México, Mexico City, Mexico; University of Agriculture Faisalabad, PAKISTAN

## Abstract

Approaches to the estimation of shadow prices generally assume that all but one market function correctly. However, multiple market failures are common in developing countries. We present a theoretical model and an empirical strategy to estimate the shadow price of a subsistence good in an economy where labor markets fail. Our results show that: 1) among subsistence producers, the shadow price of this good must be greater than or equal to the market price, and equal to it for surplus growers; and 2) current methods create biases when the otherwise-perfect-markets assumption is violated. The propositions are tested using a representative survey for rural Mexico. We find that the shadow wage is below that of the market (MXN $93.2/day vs. MXN $132.3/day), and that the shadow price for subsistence corn is over ten times greater than its market price (MXN $32.37/kg vs. MXN $3.19/kg). Unbiased shadow price estimates for subsistence goods help to overcome the limitations of current income poverty measures: their overestimation of the purchasing power of subsistence households and their underestimation of the value of subsistence goods. In rural Mexico, current practice underestimates the population in food poverty by 2%; an additional 9% has income above the poverty line yet fail to meet the utilization dimension of food security.

## Introduction

The complex behavior of households in rural developing areas is due only in part to the increasing diversification of their income sources [1]. It also owes much to the fact that their activities depend on productive factors and inputs that they own, rent, or sometimes borrow [2]. It is even difficult to estimate the net income of households that consume all or part of the final goods they produce [2]. In principle, in properly functioning markets, the amount that is produced or consumed is a function of market prices. Yet when there are market failures, these decisions depend on endogenous, non-observable shadow values that may differ substantially from market prices [3]. Importantly, when income depends on shadow prices, they not only affect the level of household poverty, but also the consequences that follow from its inaccurate measurement.

Shadow and market prices can differ in various types of market failure, including those involving transaction costs, which are often linked to poor transportation. Such costs make it difficult to buy and sell goods. As these transaction costs rise, self-sufficiency ultimately becomes the best option, and gaps develop between the shadow and market prices [3]. A gap in the price of output is also to be expected when markets do not recognize qualities valued by subsistence households [4–6]. For instance, corn farmers in Oaxaca, Mexico reportedly prefer their own grain to that sold in the market when they prepare special meals or herbal medicines, yet they also grow the crop to preserve their culture and traditions [7]. Another seemingly common market failure is the imperfect substitution of family and hired labor that encourages household members to employ themselves at home rather than in the market [8, 9]. Both circumstances tend to create a gap between market and shadow wages. This gap may also arise when limited demand for hired labor creates involuntary unemployment [10, 11]. Simultaneous market failures may well account for multiple shadow prices in most rural settings in the developing world, with implications for poverty and its measurement.

In this article we propose a theoretical model of household behavior in a mixed subsistence/commercial economy with two market failures. Failures are assumed due to: i) the highly heterogeneous quality of subsistence goods and ii) labor unemployment. Based on the theoretical solution to this problem we propose a protocol to estimate shadow wages using nationally representative data for Mexican rural households, and we use these results to estimate the shadow price of corn grown in monoculture by this population. This latter restriction allows us to control for the value of other crops often grown in polyculture in Mexico in our estimates of corn production. Finally, we use shadow prices for corn to revise current federal poverty measures in two ways, which are also relevant to other developing countries where government programs depend on income-poverty lines based on the market price of a certain food basket. More specifically, first, we find subsistence households that are currently classified as non-poor although they cannot afford the basket. They are not considered poor because they can afford to buy the reference basket, including the recommended volume of corn; i.e., their income is above the line that defines food insecurity. However, they choose not to buy but grow costlier corn because its quality is much superior to the market’s. Then we find households currently classified as poor whose net income is actually above the basket’s market price, due to the high value of home-grown corn. While they can afford the basket, they do not have a diverse diet. Opting to consume an “excess” of costly corn, they still fail the utilization dimension of food security and remain insecure.

This article is divided into five sections, beginning with a review of the literature on shadow prices and their estimation. The second section develops the theoretical model, describes first-order conditions, and derives equations to calculate both the shadow price of a subsistence good and the shadow wage of family labor. Based on these equations, the third section makes an econometric estimate of the shadow price of corn and the shadow wage in rural Mexico. The fourth section addresses the implications for poverty and its measurement. A final section presents our conclusions.

## Section 1: Shadow values in developing economies

The empirical literature on shadow values consists mostly of studies of the value of agricultural inputs or produce. These studies have employed the market price of both produce and inputs to estimate the shadow value of other agricultural inputs and productive factors. These include estimates of the shadow wage of family labor (the opportunity cost of household leisure) and occasional estimates of full income [8–12]. These estimates have been used jointly to estimate the supply of rural labor [8–12]. Studies have assumed that labor markets fail either due to unemployment [10–12] or to the imperfect substitution of family and hired labor [8, 9]. Assuming the latter, Jacoby [8] equated the shadow wage of farmers in Peru to the marginal value product of family labor in agricultural production. Skoufias [9] used the same approach to estimate the shadow wage of farmers in India, correcting for possible bias by controlling for the endogeneity of production inputs due to unobserved time-invariant factors. Recognizing the existence of inefficiency in the allocation of family labor, Barrett, Sherlund [13] generalized this approach by considering other possible failures in the labor market, such as risks and uncertainty, transaction and/or research costs, and preferences regarding labor allocation.

Among studies of this kind, Sonoda and Maruyama [11] found that with unemployment, the response of farmers’ shadow wages to prices accounted for the downward slope of the rice supply in Japan. The change in the price of rice has two effects on its supply: the first is direct and positive, and coincides with the situation without unemployment, while the second corresponds to the change in shadow wage, is indirect and negative, and counters the first effect. Also assuming the presence of unemployment, Stabridis-Arana [12] found that in 2007, the shadow wage of Mexican farm-households practicing agriculture or raising livestock was below the market wage. Le [10] estimated the shadow wage of agricultural landowners in Vietnam based on the market price of another input, fertilizer. Unlike his predecessors, avoiding estimation, he assumed a semi-parametric production function to find shadow wages and income, estimating these along with an equation for the supply of labor, based on a system of two equations solved using the generalized method of moments.

Few studies have dealt with the shadow price of productive factors other than labor. Magnan, Larson [14] estimated the shadow price of non-market stubble (an input in cattle raising in Morocco) using the market price of straw, a market input. Apparently, only Arslan and Taylor [4] have estimated the shadow price of produce, in this case corn in Mexico, using market wages. Their choice of input was based on their inability to reject the presence of full employment in rural Mexico in 2003. However, there is now evidence of widespread unemployment there in 2007 [12].

## Section 2: Theoretical model

To further develop a line of theory begun by Jacoby [8] and Skoufias [9] and leading to Arslan and Taylor [4], we derive an equation in this section for the shadow price of a subsistence good *s* (like corn in Mexico) in a mixed subsistence/commercial economy where there is unemployment [10, 11]. We begin by describing the decision-making of a household that can sell but not buy good *s* at market prices, presumably because of differences in quality among suppliers. The household also produces a commercial good *c* (like livestock in Mexico) that is both easily bought and sold in the market. We first describe the allocation of the household’s time endowment—i.e., time available to adult members—among its various activities. We then focus on its production of goods *s* and *c*, and we then characterize its consumption.

The household distributes its time (*T*) among: i) labor offered in the market (*M*); ii) family labor used at home in the production of the subsistence (*F*_*s*_) and/or commercial good (*F*_*c*_), and iii) time dedicated not to labor but leisure (*L*), so that *T* = *L* + *M* + *F*_*s*_ + *F*_*c*_. Following Le [10] and Sonoda and Maruyama [11], the household’s supply of labor in the market cannot exceed an upper bound *δ*, due to unemployment, so that *M* ≤ *δ*. The commercial good *c* is not consumed at home. Its production requires family labor (*F*_*c*_), hired labor (*H*_*c*_), and fixed production factors (*K*_*c*_), i.e., *Q*_*c*_ (*F*_*c*_, *H*_*c*_, *K*_*c*_). Production of the subsistence good *s* requires these same factors, i.e., *Q*_s_(*F*_*s*_, *H*_*s*_, *K*_*s*_), but its output can be devoted either to home consumption (*C*_*s*_) or to sale (*Q*_s_ − *C*_s_).

The household’s utility is a function of the consumption of a composite purchased good *C*_*x*_, *C*_*s*_, leisure (*L*), and a vector (*B*) of household characteristics that influence its preferences: *U*(*C*_*x*_, *C*_*s*_, *L*; *B*). Substituting *L* in *U(·)* using the time constraint, the household’s consumption choices (*C*_*x*_, *C*_*s*_), production decisions (*H*_*s*_, *H*_*c*_), and time allocation (*M*, *F*_*s*_, *F*_*c*_), we solve the following optimization problem:

$$Max\;U\left[C_{x};C_{s};T-M-F_{s}-F_{c};B\right]$$ (1)

subject to

$$p_{x}C_{x}=p_{c}Q_{c}\left(F_{c},H_{c},K_{c}\right)-wH_{c}+w^{m}M+V+p_{s}\left(Q_{s}\left(F_{s},H_{s},K_{s}\right)-C_{\text{s}}\right)-wH_{s}$$ (2)

$$C_{s}\leq Q_{s}\left(F_{s},H_{s},K_{s}\right)$$ (3)

M≤δ, (4)

where *p*_s_, *p*_c_, and *p*_*x*_ are the market prices of the subsistence and commercial goods produced by the household and of other purchased goods, respectively, *w* is the wage for hired work by the household, *w*^*m*^ denotes the market wage the household receives for its work, *M*, and *V* is the household’s exogenous income (consisting of public and/or private transfers).

The Kuhn-Tucker conditions generate the following results (see S1 Appendix):

$$w^{*}=p_{\text{c}}\frac{\partial Q_{c}\left(F_{c},H_{c},K_{c}\right)}{\partial F_{c}}$$ (5)

$$w=p_{\text{c}}\frac{\partial Q_{c}\left(F_{c},H_{c},K_{c}\right)}{\partial H_{c}}$$ (6)

$$w^{*}=p_{s}^{*}\frac{\partial Q_{\text{s}}\left(F_{s},H_{s},K_{s}\right)}{\partial F_{s}}$$ (7)

$$w=\;p_{s}^{*}\;\frac{\partial Q_{\text{s}}\left(F_{s},H_{s},K_{s}\right)}{\partial H_{s}}$$ (8)

$$p_{s}^{*}=\frac{w^{*}}{\frac{\partial Q_{\text{s}}\left(F_{s},H_{s},K_{s}\right)}{\partial F_{s}}}$$ (9)

$$w^{*}=w^{m}-\frac{\mu }{\lambda }$$ (10)

$$p_{s}^{*}=p_{\text{s}}+\frac{\mu_{s}}{\lambda }$$ (11)

where *λ*, *μ*_*s*_, and *μ* (with *μ*_*s*_, *μ* ≥ 0) are the multipliers associated with Eqs (2), (3) and (4), while w* and $p_{s}^{*}$ are the shadow values of family labor and good *s*, respectively.

Eqs (5) and (7) demonstrate that, notwithstanding market imperfections, production decisions occur when the price of each production factor equals the value of its marginal product. This condition aligns with the classical assumptions of perfect market scenarios.

The intuition behind Eq (5) is as follows. If the household allocates a level of family labor (*F*_*c*_) to produce good c in such a way that $w^{*}<p_{\text{c}}\frac{\partial Q_{c}}{\partial F_{c}}$, then the cost of investing an additional unit of family labor is less than the benefit obtained. Consequently, the household will increase its demand for *F*_*c*_, leading to two implications. Firstly, the shadow price of family labor rises (↑ w*), as it becomes a scarce resource. Secondly, a decline in the marginal product occurs causing its value to decrease (${↓p}_{c}\frac{\partial Q_{c}}{\partial F_{c}}$). These two quantities approach each other, and this process continues until equality is reached (Eq (5)). The logic for the opposite case ($w^{*}>p_{\text{c}}\frac{\partial Q_{c}}{\partial F_{c}}$) is similar.

The rationale behind Eq (7) can be explained as follows. When a level of family labor (*F*_*s*_) is selected in such a way that $w^{*}<p_{s}^{*}\frac{\partial Q_{\text{s}}\left(F_{s},H_{s},K_{s}\right)}{\partial F_{s}}$, it triggers an increase in its demand, leading to three effects. Similar to the previous scenario, we observe both ↑w* and $↓\frac{\partial Q_{s}}{\partial F_{s}}$. Additionally, the heightened family labor input leads to increased production, creating a surplus of the grain for the household, which, in turn, causes a decrease in its price $↓p_{s}^{*}$. In essence, ↑w* and ${↓p}_{s}^{*}\frac{\partial Q_{s}\left(F_{s},H_{s},K_{s}\right)}{\partial F_{s}}$ bring these quantities closer together, and this iterative process continues until equilibrium is reached. The opposite situation follows a similar logical pattern.

Eq (5) shows that the shadow wage, w*, is equal to the value of the marginal product of family labor employed in the production of good *c*. Eq (9) shows that the shadow price of good *s* is equal to the shadow wage w* divided by the marginal product of family labor dedicated to *s*. It is clear, from Eqs (10) and (11), given that λ=(1px)(∂U∂Cx)>0 and the definitions of *μ* and *μ*_*s*_, that the shadow price of *s* is greater than or equal to its market price, and that the shadow wage is less than or equal to the market wage (*w*^*m*^) (The intuition for this result can be found in Sonoda and Maruyama [11]). For the shadow wage to coincide with its market counterpart, the complementary slackness condition requires full employment: i.e., *μ*(*M* − *δ*) = 0. Similarly, the necessary condition for the shadow and market prices of good s to coincide is that the household sell any amount of that good, i.e., *μ*_*s*_(*C*_*s*_ − *Q*) = 0. Two implications of observing imperfections in the labor and subsistence good markets simultaneously follow:

Proposition 1. If the price of a subsistence good is used to estimate the marginal value product of family labor employed in its production, estimates of the household’s shadow wage (as per Eq 7) will be biased downwards whenever this good’s market fails (i.e., $p_{s}^{*}\neq p_{s}$). Its demonstration follows from Eqs (7) and (11), and the fact that *μ*_*s*_ ≥ 0_∎_.
Proposition 2. If labor markets fail (i.e., w* ≠ *w*^*m*^), estimates of the shadow price of a subsistence good based on market rather than shadow wages (as per Eq 9) will be biased upwards. The proof comes from Eqs (9) and (10), and the fact that *μ* ≥ 0_∎_.

## Section 3: Application to rural Mexico

**Fig 1:**
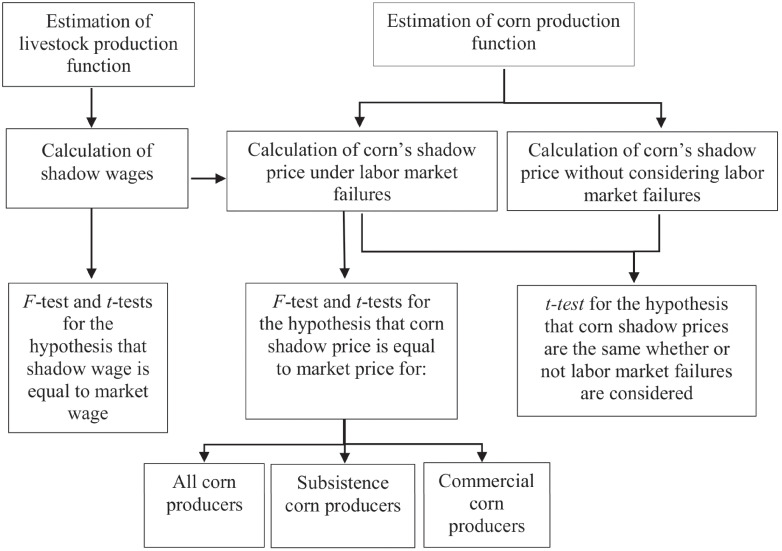
Flow diagram showing estimation procedures for the shadow value of corn and family labor, and related hypothesis testing.

In this section, we use solutions to the theoretical model and nationally representative data for Mexican rural households to estimate the shadow wage and price of corn in this population. Corn is defined as the subsistence good (*s*), while livestock production represents the commercial good (*c*). We then use these results to test Proposition 2, derived above. We obtain the shadow price of corn in five steps (Fig 1): i) we estimate a production function for livestock, which we use ii) to derive the marginal value product of family labor raising livestock: its shadow wage (Eq 5); iii) we estimate a production function for corn iv) and use both ii) and iii) to calculate the shadow price of corn (Eq 9) under two scenarios, full employment and unemployment. Finally, v) we test whether the estimated shadow and market prices differ econometrically. Hypothesis tests are conducted to determine the relation of the shadow wage and price of corn for rural households to their counterparts in the market. The relationship between the two alternative estimates of the shadow price of corn reported in step iv) is also tested.

### Data

Our data for the Mexican rural population come from the 2007 National Survey of Rural Households in Mexico (*Encuesta Nacional a Hogares Rurales de México*, ENHRUM). The ENHRUM contains information on the income and expenditure of 1,765 households and their production, consumption, employment, credit, assets, and savings. The survey is representative of the rural population nationwide and in its five economic regions. See S1 Table for the descriptive statistics of variables used and http://investigaciones.colmex.mx/enhrum/ for the survey questionnaire and data.

### Estimates of the rural shadow wage

The model’s solution sets the shadow wage equal to the marginal value product of family labor raising livestock (Eq 5), which can be derived from its marginal product in this activity, after estimating the production function for livestock. We estimate this function by correcting for the possible sample selection bias generated by the self-selection of those raising livestock [12]. Specifically, we use a production model consisting of a participation equation and a production equation [15–17]. The participation equation is given by:

$$d_{i}=\left\{\begin{array}{l} 1\;if\;d_{i}^{*}=\gamma x_{i}+v_{i}>0\\ 0\;if\;d_{i}^{*}=\gamma x_{i}+v_{i}\leq 0 \end{array}\right.$$ (12)

where $d_{i}^{*}$ is unobserved except for its sign, ***x***_***i***_ is a vector of variables representing the household’s characteristics that shape its decision to raise livestock, ***γ*** is a vector of parameters, and *v*_*i*_ is an unobservable error term. Finally, *d*_*i*_ is a dichotomous variable that takes the value 1 if the *i*th household engages in livestock raising and 0 if it does not.

When *d*_*i*_ = 1, the observed production equation is given by:

$$y_{i}=\beta \omega_{i}+\varepsilon_{i}$$ (13)

where *y*_*i*_ is the logarithm of the value of livestock production, ***β*** is a vector of parameters, ***ω***_***i***_ is a vector of known variables that determine output, including household characteristics, inputs, and technology, and *ε*_*i*_ is an error term. This specification assumes a Cobb-Douglas production function.

Parameters in (12) and (13) are estimated assuming that (*v*_*i*_, *ε*_*i*_) are jointly normal and using maximum likelihood estimation. Results for both the selection and production equations are presented in columns (a) and (b) of Table 1. The likelihood ratio test rejects the hypothesis of no selection bias (*p* < 0.01), justifying our model. The shadow wage of family labor is calculated based on Eqs (5) and (13), following Jacoby [8], using the expression:

$$w^{*}=\frac{\hat{\beta_{i}}*e^{\hat{y_{i}}}}{L_{i}}$$ (14)

where *L*_*i*_ is the number of days of family labor employed raising livestock, $\hat{\beta_{l}}$ is the coefficient associated with the log of family labor, log(*L*_*i*_), in Eq (13), and $\hat{y_{i}}$ is the logarithm of the predicted value of livestock output.

**Table 1:** Livestock production estimates corrected for sample selection bias.

Independent Variable	Log Livestock Production (thousands of MXN $)	Probability of Raising Livestock
Years of Education of Household Head	0.02	0.01
	(0.02)	(0.01)
Years of experience of household head (age minus five minus years of schooling)	0.01	0.03**
	(0.02)	(0.01)
Experience squared	-0.00	-0.00***
	(0.00)	(0.00)
Household size	0.02	0.00
	(0.01)	(0.01)
Sex of household head (1 = Male, 0 = Female)	0.27*	0.22**
	(0.14)	(0.11)
Indigenous household (1 = Yes, 0 = No)	-0.15	0.17
	(0.13)	(0.12)
Central region (1 = Yes, 0 = No)	-0.05	-0.02
	(0.14)	(0.13)
Center-West region (1 = Yes, 0 = No)	0.02	-0.12
	(0.16)	(0.14)
Northwest region (1 = Yes, 0 = No)	0.44**	-0.63***
	(0.21)	(0.14)
Northeast region (1 = Yes, 0 = No)	0.36*	-0.11
	(0.19)	(0.15)
Value of own food for livestock (thousands of MXN $)	0.02***	
	(0.00)	
Expenditure in food for livestock (thousands of MXN $)	0.01***	
	(0.00)	
Expenditure on rent of grazing land (thousands of MXN $)	0.11***	
	(0.03)	
Contingent value of own pastures (thousands of MXN $)	0.12***	
	(0.03)	
Expenditure on vaccines, vitamins, and insemination for livestock (thousands of MXN $)	0.28***	
	(0.05)	
Veterinary expenses (thousands of MXN $)	-0.29**	
	(0.14)	
Log of the days of family labor spent on livestock activity	0.49***	
	(0.03)	
Log of the days of hired labor used on livestock activity	0.04	
	(0.07)	
Household has installations such as corrals, stalls, or stables (1 = Yes, 0 = No)	0.43***	
	(0.10)	
Household uses machinery for animal breeding and fattening such as sprinklers or drinkers (1 = Yes, 0 = No)	0.53***	
	(0.19)	
Expenditure on transportation for buying/selling animals (thousands of MXN $)	0.04	
	(0.04)	
Access to credit for livestock (1 = Yes, 0 = No)	1.18***	
	(0.33)	
Income transfers to farmers from PROGRAN (thousands of MXN $) ^a^	0.03	
	(0.03)	
Income transfers to farmers from OPORTUNIDADES (thousands of MXN $) ^a^	0.01	0.04**
	(0.02)	(0.02)
Income transfers to farmers from PROCAMPO (thousands of MXN $) ^a^	0.04***	0.04***
	(0.01)	(0.01)
Crop producer in 2002 (1 = Yes, 0 = No)		0.34***
		(0.09)
Livestock producer in 2002 (1 = Yes, 0 = No)		0.94***
		(0.08)
Constant	5.49***	-1.25***
	(0.47)	(0.33)
Observations	802	1,405

*** *p* < .01,

** *p* < .05,

* *p* < .1

Source: Authors’ elaboration.

^a^ PROGRAN, PROCAMPO, and OPORTUNIDADES are government programs that support livestock production, crop production, and social development, respectively.

Note: Single asterisk (*), double asterisks (**), triple asterisks (***) indicate statistical significance at the 10%, 5%, and 1% levels, respectively.

The average shadow wage of family labor is calculated at MXN $ 93.2/day. A difference of means test shows that this figure is significantly lower than the market wage, MXN $132.3 (*p* < 0.01). We conduct an additional test for the presence of failures in the labor market, following Jacoby [8] and Arslan and Taylor [4], by modeling the estimated shadow wage for household *i*, ${\hat{\rho }}_{i}$, as a linear function of the market wage for *i*, *ρ*_*i*_:

$${\hat{\rho }}_{i}=\alpha +\gamma \rho_{i}+\tau_{i}$$ (15)

where *τ*_*i*_ is an error term. An *F*-test is then performed to determine whether the shadow and market wages are the same, i.e., *H*_0_: *α* = 0 and *γ* = 1. *T*-tests are conducted to determine specifically whether to reject *H*_0_: *α* = 0 and *H*_*0*_: *γ* = 1. Estimates for *α* and *γ* and *p*-values (reported in the first row of Table 2) reject the equality of shadow and market wages: they support the presence of failures in the labor market, as reported by Stabridis-Arana [12].

**Table 2:** Statistical tests comparing shadow and market prices of corn.

Null hypothesis (*H*_*0*_)		γ	α	*t*-test		*F*-test	Conclusion of *t*- and *F*-tests^a^
				*p*-value: $H_{0}^{\prime }$: α = 0	*p*-value $H_{0}^{\prime }$: γ = 1	*p*-value $H_{0}^{\prime }$: α = 0, γ = 1	
Shadow and market wages are equal		0.05	87.09	0.00	0.00	0.00	Reject *H*_*0*_ at a significance level of 1%
Shadow and market prices of corn are equal for:	All corn producers	1.22	28.48	0.02	0.95	0.00	Reject *H*_*0*_ at a significance level of 5%
	Commercial corn producers	4.90	8.94	0.27	0.14	0.00	Do not reject *H*_*0*_
	Subsistence corn producers	-2.10	43.49	0.01	0.53	0.00	Reject *H*_*0*_ at a significance level of 5%
Corn shadow prices are the same whether or not labor market failures are considered		0.22	26.75	0.00	0.00	0.00	Reject *H*_*0*_ at a significance level of 1%

Source: Authors’ elaboration.

^a^ As in [4], the null hypothesis is rejected only when the *F*-test and at least one of the two *t*-tests reject it.

Note: In all cases the dependent variable of the regression model is the shadow wage or price, and in the last row, the dependent variable is the corn shadow price estimated without considering the labor market failures.

### Estimates of the shadow price of corn

The presence of unemployment in rural Mexico implies that estimates of the shadow price of corn that do not account for this market failure will be biased (Proposition 2). To test this proposition, we propose here an estimation method that accounts for failures in both markets simultaneously, and we then present estimation results and hypothesis tests.

Since the solution to the model sets the shadow price of corn as equal to the ratio of the shadow wage and the marginal product of family labor in corn production (Eq 9), the first step requires estimating a production function for corn. As for livestock, this function is estimated using two equations (participation and production) to correct for a possible selection bias. The participation equation is given by:

$$d_{i}^{m}=\left\{\begin{array}{c} 1\;if\;d_{i}^{*,m}=\gamma^{m}x_{i}^{m}+v_{i}^{m}>0\\ 0\;if\;d_{i}^{*,m}=\gamma^{m}x_{i}^{m}+v_{i}^{m}\leq 0 \end{array}\right.$$ (16)

where only the sign and not the magnitude of $d_{i}^{*,m}$ is known, while $x_{i}^{m}$ is a vector of variables that influence the household’s decision to plant corn, and $v_{i}^{m}$ is an error term. $d_{i}^{m}$ is a dichotomous variable that takes the value of 1 if the household plants corn and 0 otherwise.

The production equation observed when $d_{i}^{m}=1$ is defined in the following manner:

$$y_{i}^{m}=\beta^{m}\omega_{i}^{m}+\varepsilon_{i}^{m}$$ (17)

where $y_{i}^{m}$ is the logarithm of household *i*’s corn output in kilograms, $\omega_{i}^{m}$ is a vector of variables that determine production, including household characteristics, inputs, and technology, and $\varepsilon_{i}^{m}$ is an error term.

Parameters *γ*^*m*^ and *β*^*m*^ in Eqs (16) and (17) are estimated using maximum likelihood. Estimation results (reported in Table 3) reject the absence of sample bias (*p* < 0.01) and justify the use of the methodology. The shadow price of corn is calculated based on Eqs (9) and (17) and the shadow wage previously obtained, according to the following equation:

$$p_{s}^{*}=\frac{w^{*}}{\frac{\hat{\beta_{l}^{m}}*e^{\hat{y_{i}}}}{L_{i}}}$$ (18)

where *L*_*i*_ is the number of days of family labor employed to produce corn, $\hat{\beta_{l}^{m}}$ is the estimated coefficient of log(*L*_*i*_) in Eq (17), and $\hat{y_{i}}$ is the predicted logarithm of corn production.

**Table 3:** Production function estimates for corn corrected for simple selection bias.

Independent Variable	Log of Corn Production (kg)	Probability of Planting Corn
Years of education of household head	0.03	-0.04**
	(0.03)	(0.02)
Years of experience of household head (age minus five minus years of schooling)	0.01	-0.01
	(0.02)	(0.02)
Experience squared	-0.00	0.00
	(0.00)	(0.00)
Household size	-0.01	0.01
	(0.02)	(0.02)
Sex of household head (1 = Male, 0 = Female)	0.08	0.38**
	(0.23)	(0.16)
Indigenous Household (1 = Yes, 0 = No)	0.00	0.25*
	(0.18)	(0.13)
Center region (1 = Yes, 0 = No)	0.27	0.15
	(0.19)	(0.14)
Center-west region (1 = Yes, 0 = No)	0.86***	-0.10
	(0.25)	(0.17)
Northwest region (1 = Yes, 0 = No)	1.38***	-0.30
	(0.39)	(0.22)
Northeast region (1 = Yes, 0 = No)	1.75***	-0.73***
	(0.39)	(0.23)
Expenditure in fertilizers, manure, and other agrochemicals (thousands of MXN $)	0.03**	
	(0.01)	
Use of native seeds (1 = Yes, 0 = No)	-0.22	
	(0.17)	
Machinery used before harvest (other than animal traction)	0.42***	
	(0.15)	
Use of animal traction (1 = Yes, 0 = No)	-0.06	
	(0.12)	
Machinery used during harvest	-0.06	
	(0.21)	
Access to irrigation (1 = Yes, 0 = No)	0.63***	
	(0.15)	
Log of the amount (days) of family labor spent to cultivate	0.13**	
	(0.05)	
Log of the amount (days) of hired labor used to cultivate	0.08*	
	(0.05)	
Log of cropped area	0.61***	
	(0.07)	
Credit access to cultivate (1 = Yes, 0 = No)	1.01***	
	(0.30)	
Income transfers to farmers from PROCAMPO (thousands of MXN $)	0.02	0.02*
	(0.02)	(0.01)
Income transfers to farmers from PROGRAN (thousands of MXN $)	0.05	-0.03
	(0.09)	(0.05)
Income transfers to farmers from OPORTUNIDADES (thousands of MXN $)	0.03	0.03
	(0.04)	(0.03)
Average distance from house to plot (km)		-0.03**
		(0.01)
Livestock producer in 2002 (1 = Yes, 0 = No)		0.18
		(0.11)
Crop producer in 2002 (1 = Yes, 0 = No)		0.13
		(0.11)
Constant	6.27***	-0.41
	(0.74)	(0.48)
Observations	300	754

Source: Authors’ elaboration.

The average shadow price of corn is calculated at MXN $32.37/kg, which is significantly above its market price, MXN $3.19/kg (*p* < 0.01). We test for the presence of failures in the corn market, that is, whether the shadow and market prices differ, following Jacoby [8] and Arslan and Taylor [4]. Test results presented in the second row of Table 2, corresponding to the linear regression of shadow prices on market prices, reveal failures in the corn market (*p* = 0.02).

### Empirical tests of theory

In this section we derive two sets of inferences from test results. The first set concerns the association of estimates of the shadow and market prices of corn. According to theory, the shadow and market prices will be the same for households selling corn, yet these prices will differ for those that produce exclusively for their own consumption. Adapting Jacoby [8] and Arslan and Taylor [4] to validate these inferences, we regress shadow prices on their market counterparts—i.e., shadow price (i.e., $p_{s}^{*}$) = α + γ* market price (i.e., p_s_)—for two groups of interest, namely subsistence and surplus farms, as only the latter sell grain. An *F*-test and two *t*-tests are used to determine whether *α* = 0 and *γ* = 1. Findings are reported in rows 3 and 4 of Table 2.

The *F*-test rejects the equality of the shadow and market prices for corn from surplus farms, yet the individual *t*-tests show otherwise: the two prices do not differ significantly (row 3, Table 2). For subsistence farms, which sell no grain, both *F-* and *t*-tests reject the null hypothesis (*p* = 0.02, row 4); shadow and market prices differ significantly. In sum, as theory would have it, subsistence farmers in rural Mexico do not respond directly to the market price of corn, as reported by Dyer [5].

The second set of inferences is based on a comparison of estimates of the shadow price of corn under contrasting assumptions: with and without labor market failures. In theory, price estimates that wrongly assume full employment will be biased upwards. Indeed, we reject the equality of estimates (*p* < 0.01; row 5 of Table 2). A test of means shows that estimates wrongly assuming full employment are higher, on average, than those accounting for unemployment (*p* = 0.05), validating Proposition 2. It’s worth noting that, according to Eq (10), under full employment, w* = *w*^*m*^. Therefore, when calculating the shadow price of corn without considering labor market failures as per Eq (18), w* is taken as the wage received by the household for its work in the market (*w*^*m*^).

## Section 4: Implications for income-based poverty measures

Income-based measures of poverty are mostly simple functions of differences between household income and an exogenous poverty line or threshold, itself often a function of the cost of a “basic consumption basket,” as in Mexico [18]. Notably, they include the three Foster–Greer–Thorbecke (FGT) metrics of poverty: a) the headcount ratio (i.e., the fraction of the population whose income is below the poverty line), b) the poverty gap, and c) the severity of poverty [19]. Studies addressing the limitations of income-based measures include Alkire and Santos [20], Barrett [21], Ravallion [22] and Sen [23]. Among the major drawbacks has been a failure to account for the considerable obstacles facing vulnerable sectors of the population (e.g., pregnant women) when they try to convert income into functionings [20]. At times, where policy depends exclusively on poverty headcounts, as in Mexico, public support has tended to focus disproportionately on easy-to-redeem households (i.e., those closest to the poverty line) at the expense of the poorest, creating “jumps” in the distribution of income around the line [22].

The response to these shortcomings thus far has been the development of multidimensional methodologies meant to complement rather than substitute for income-based measures [20], which remain widely used and relevant if susceptible to improvement. Income-based measures do not yet account for the effect of market failures on poverty and its measurement. Both commercial and subsistence goods are generally valued at market prices, incurring two relatively common errors with opposite effects on the resolution of any analysis, as described below. The overall effect of using market rather than shadow prices is thus an empirical question with multifactorial causality, dependent on characteristics of the population as much as on the economy or the composition of the basket.

### Measures of poverty in Mexico

Official federal government estimates of the prevalence of poverty in Mexico are the responsibility of the National Council for the Evaluation of Social Development Policy (Consejo Nacional de Evaluación de la Política de Desarrollo Social, CONEVAL), which recognizes poverty as a multidimensional condition yet places overwhelming emphasis on food poverty, whose sole determinant is income [24–27]. CONEVAL’s poverty estimates thus address almost exclusively the access dimension of food security, largely neglecting its utilization and stability dimensions [28]. To this end it has formally developed and regularly updates a protocol to estimate the incidence of food poverty across the country, reporting the number and prevalence of food-insecure households in both urban and rural areas, as well as across states and municipalities. Its estimates have implications for the eligibility of households to receive social development assistance [25]. A central aspect of CONEVAL’s work is thus to establish the income threshold that places households neatly within a certain food security status: either in food poverty or above it [25, 29]. The threshold is defined as the amount necessary to purchase a basic nutritious basket, an ad hoc expenditure pattern consistent with the officially recommended daily caloric and nutrient intake [30]. Ostensibly, the basket’s contents reflect actual expenditure among a specific segment of the urban and rural population; more specifically, the income quintile surrounding those households in the National Household Income and Expenditure Survey (ENIGH) sample that consume the exact daily recommended caloric intake [29]. However, CONEVAL revises this group’s consumption until the reference basket satisfies the recommended intake of other macro and micro nutrients [29]. The basket assumes a consumption of 70 g of corn and 218 g of tortillas per person per day, which represents a total of 226 g of corn. The basket’s value is next defined as the “efficient” (i.e., minimum) monetary income necessary to avoid food poverty, which routinely considers non-market income and expenditure at market prices [29]. Ultimately, CONEVAL uses ENIGH survey expansion factors to extrapolate the incidence of poverty among sample households into nationwide estimates.

We illustrate the implications of valuing subsistence corn production in Mexico at market prices by considering the counterfactual accounting for its shadow value, and reporting differences in the first FGT metric of poverty for Mexican rural areas. We closely follow CONEVAL’s guidelines for estimation but base the analysis on the ENHRUM 2007 survey sample rather than on that of the ENIGH, which does not contain all the information used here to estimate the shadow price of corn (see below). To create an appropriate benchmark, we calculate the per capita income (adjusted for economies of scale and equivalence scales [26]) of every household in the ENRHUM sample, using market prices, and we compare the resulting distribution against the official poverty line, i.e., the market value of the basket. We then repeat the entire protocol using shadow prices of corn instead of market prices, and revise individual household income and the cost of the basket. Consistent with the results reported above, the shadow and market prices are the same for surplus farms but significantly different for subsistence farms (Table 2). The basket’s entire corn allowance—representing 66% of the average consumption of corn, by volume, among subsistence households in rural Mexico (own data)—is valued at shadow prices, since evidence links positive income shocks there to the expansion of subsistence agriculture, particularly among smallholder and landless households with no surpluses [5, 31, 32].

Only 88% of the rural population practicing subsistence agriculture—i.e., the sector directly affected by the shadow prices of corn—are consistently classified as either poor or non-poor across the two methods, shadow and market pricing; another 12% must be reclassified after accounting for the shadow price of corn (Table 4). Approximately 1.8 million people, 49% of the population, are identified as food insecure in both cases. Valuing subsistence consumption of corn at market prices entails the artificial lowering of the poverty line for a significant sector of the population whose staple food is relatively costly yet of high quality. An additional 89,000 people (2%) fall below the poverty line revised to consider the shadow price of corn. These households grow relatively little corn, but when its cost is reconsidered, it is evident that they cannot afford the volume recommended in the basket.

**Table 4:** Subsistence population classified by poverty status using market and shadow prices.

Status		Shadow Prices					
		Millions of People			Percent		
		Non-poor	Poor	Total	Non-poor	Poor	Total
Market prices	Non-poor	1.451	0.089	1.540	39%	2%	42%
	Poor	0.350	1.800	2.151	9%	49%	58%
	Total	1.801	1.890	3.691	49%	51%	100%

Still, only 51% of those practicing subsistence agriculture have incomes below the revised food poverty line, while 58% are classified as poor assuming market values. What explains this apparent drop in poverty is that adjusting the value of corn entails a considerably larger increase in average household income than in the cost of the basket: 170.14% vs. 42.70%.

This outcome is ultimately tied to CONEVAL’s definition of the basket’s contents and the resulting discrepancies with diets in rural Mexico. In fact, because of these discrepancies, the revised headcount ignores another 9% of the subsistence population, 350,000 people, who must now be reclassified as non-poor although they remain food insecure. Their households are in the same situation as the basket’s reference population: despite their income reaching or exceeding the basket’s price, their estimated micro-nutrient intake is below the CONEVAL recommendation [30]. Due to their strong preference for corn, they allocate their limited resources to its production and consumption at the expense of consuming other products. This results in a deficiency of micronutrients lacking in corn, such as vitamins A, B12, D [33], and C [34]. Consequently, these households do not meet the utilization dimension of food security: sufficient energy and nutrient intake through a diverse diet [28]. Naturally, there is no evidence that they would achieve such security by abandoning a subsistence livelihood and buying and selling food in the market. Accounting for this group, the prevalence of food poverty in rural Mexico rises to 61%. The number of people consistently classified in 2007 as non-poor across methods is thus reduced to 1.45 million, 39% of the subsistence population.

## Section 5: Conclusions

Unemployment and the highly heterogeneous nature of subsistence goods are arguably common occurrences in rural developing areas around the world, reflecting the simultaneous presence of multiple, interacting market failures. Such circumstances help to explain the complex and sometimes puzzling behavior of rural households influenced by shadow prices. Since shadow and market prices can differ under various types of market failures, accurate estimates of shadow prices can have important policy implications. The model and methods presented here and their application to corn farmers in rural Mexico account for a pervasive situation in 2007: simultaneous failures in the corn and labor markets. We believe our results constitute more accurate estimates of their value than previous ones [4], which allows us to reassess the status of crucial markets in rural areas, a clear improvement upon the literature.

In the case of rural Mexico, we find that the shadow wage of family labor was significantly lower in 2007 than the market wage: MXN $ 93.2/day on average versus MXN $132.3. This fact has allowed us to derive unbiased estimates of the shadow price of corn at the time, which was MXN $32.37/kg, nearly an order of magnitude greater than the price of its market counterpart, MXN $3.19/kg. These estimates have in turn allowed us to correct errors in the measurement of rural poverty, particularly food poverty. Our estimates also reveal the grossly underestimated contribution of subsistence corn to the income of Mexican households under the status quo. Yet at the same time, this status quo underestimates the costs of subsistence output, thus overestimating the purchasing power of these households, with implications for their eligibility for federal assistance programs. Current practice thus discriminates against subsistence households in poverty.

Our estimates have additional applications not described here. For instance, accurate estimates of the shadow price of corn in Mexico could be used to explain lack of participation in programs meant to bolster household income, such as the current guaranteed price scheme for corn and other staples, the predominant agricultural program since 2019. More specifically, estimates could be used to assess the rate of exclusion of subsistence farmers both in and above poverty, that is, the percentage of those not supported by the program since their shadow value for corn exceeds guaranteed prices. Since shadow incomes are an important determinant of household labor decisions, more precise estimates should also help yield better estimates of the supply of family labor than are now available. Estimates could provide important inputs to realistic general equilibrium modeling of developing rural economies. More generally, our methods allow for the estimation of shadow prices both of products with missing markets and of products derived entirely from household labor. These imply common activities across developing rural areas, such as fetching water for domestic use, hunting wildlife, or collecting firewood.

Lastly, it’s important to mention that although the applied econometric model addresses the challenge of selection bias, the use of cross-sectional data presents significant limitations. One constraint relates to the issue of endogeneity arising from unobservable variables that are correlated with inputs, such as management ability and soil characteristics [4, 8, 10]. A commonly employed strategy to mitigate this predicament involves the use of instrumental variables. However, as emphasized by several scholars [8, 10, 35], the identification of robust and valid instruments remains intricate.

In an ideal scenario, the estimation of production functions would make use of panel data, allowing for the incorporation of models to handle time-invariant unobserved factors [4, 9]. However, it’s worth noting that even this approach is not without challenges, as it may not effectively address the complexities posed by time-varying unobservable variables [4].

Another complication arising in the estimation of production functions is the interdependence between corn production and livestock farming, which can be attributed to various factors. Crop residues often serve as livestock feed during dry seasons [36], while corn is used as poultry feed. Furthermore, cattle manure is employed to enrich the soil and improve its quality [37]. For small-scale corn and livestock producers, there is a competition for land use between forage crops and corn cultivation. Additionally, income generated from livestock can be utilized to finance inputs for corn farming.

The interdependence between corn production and livestock farming can introduce bias into production function estimates when analyzed independently, primarily stemming from the issue of simultaneity. While this article takes steps to tackle this challenge by considering the value of household-produced food, which includes corn and its derivatives provided to animals during the estimation of livestock production, the problem may still endure.

Hence, a noteworthy challenge for future research utilizing this approach lies in applying appropriate econometric methods and selecting suitable independent variables to counteract these biases effectively.

## Supporting information

S1 Table Descriptive statistics for household and plot characteristics used to estimate production functions for livestock and corn in rural Mexico. (DOCX)

S1 Appendix Solution to the theoretical model. (DOCX)
