# Supplementary material for: Estimating shadow prices in economies with multiple market failures
Source: PLoS One. 2023 Nov 6;18(11):e0293931. doi: 10.1371/journal.pone.0293931 (PMC10627447; doi:10.1371/journal.pone.0293931)
Supplement: S1 Table — (DOCX) [file pone.0293931.s001.docx]

**S1 Table. Descriptive Statistics for Household and Plot Characteristics Used to Estimate Production Functions for Livestock and Corn in Rural Mexico**

| Variables | Number of observations | Mean | Standard deviation | Min | Max |
| --- | --- | --- | --- | --- | --- |
| **Selection equation (Corn and livestock production)** |  |  |  |  |  |
| Sex of the head of the household (1=Male, 0=Female) | 1,494 | 0.86 | 0.35 | 0.00 | 1.00 |
| Household is indigenous (1=Yes, 0=No) | 1,462 | 0.20 | 0.40 | 0.00 | 1.00 |
| Years of schooling of the head of the household | 1,417 | 4.75 | 3.94 | 0.00 | 21.00 |
| Household size | 1,494 | 7.41 | 3.48 | 1.00 | 25.00 |
| Center region (1=Yes, 0=No) | 1,494 | 0.22 | 0.41 | 0.00 | 1.00 |
| Center-West region (1=Yes, 0=No) | 1,494 | 0.21 | 0.41 | 0.00 | 1.00 |
| North-West region (1=Yes, 0=No) | 1,494 | 0.19 | 0.40 | 0.00 | 1.00 |
| North-East region (1=Yes, 0=No) | 1,494 | 0.16 | 0.37 | 0.00 | 1.00 |
| Income transfers to farmers from PROGRAN (thousands of MX$) | 1,494 | 0.08 | 1.15 | 0.00 | 32.00 |
| Income transfers to farmers from PROCAMPO (thousands of MX$) | 1,494 | 1.21 | 3.48 | 0.00 | 43.30 |
| Income transfers to farmers from OPORTUNIDADES (thousands of MX$) | 1,494 | 0.77 | 1.90 | 0.00 | 24.49 |
| Livestock producer in 2002 (1=Yes, 0=No) | 1,765 | 0.56 | 0.50 | 0.00 | 1.00 |
| Crop producer in 2002 (1=Yes, 0=No) | 1,765 | 0.41 | 0.49 | 0.00 | 1.00 |
| Years of experience of the head of the household (age minus ﬁve minus years of schooling) | 1,417 | 43.63 | 17.22 | 7.00 | 93.00 |
| Experience-squared | 1,417 | 2,200.20 | 1,623.27 | 49.00 | 8,649.00 |
| Average distance from house to plot (km) | 797 | 3.34 | 4.23 | 0.00 | 50.00 |

**S1 Table. (continued)**

| Variables | Number of observations | Mean | Standard deviation | Min | Max |
| --- | --- | --- | --- | --- | --- |
| **Livestock production** |  |  |  |  |  |
| Household has installations such as corrals, stalls or stables (1=Yes, 0=No) | 848 | 0.58 | 0.49 | 0.00 | 1.00 |
| Household use machinery for animal breeding and fattening as sprinklers or drinkers (1=Yes, 0=No) | 848 | 0.06 | 0.25 | 0.00 | 1.00 |
| Value of own food for livestock (thousands of MX$) | 848 | 3.77 | 12.11 | 0.00 | 175.38 |
| Expenditure in food for the cattle (thousands of MX$) | 848 | 5.69 | 16.55 | 0.00 | 209.96 |
| Expenditure due to rent of land for grazing (thousands of MX$) | 848 | 0.21 | 1.55 | 0.00 | 22.50 |
| Contingent value of own pastures (thousands of MX$) | 848 | 0.34 | 1.94 | 0.00 | 36.50 |
| Expenditure in vaccines, vitamins and insemination for the cattle (thousands of MX$) | 848 | 0.41 | 1.29 | 0.00 | 17.50 |
| Veterinary spending (thousands of MX$) | 848 | 0.07 | 0.39 | 0.00 | 5.05 |
| Expenditure in transportation for buying/selling animals (thousands of MX$) | 848 | 0.05 | 1.04 | 0.00 | 30.00 |
| Credit access for livestock (1=Yes, 0=No) | 848 | 0.02 | 0.13 | 0.00 | 1.00 |
| *Log* of the amount (days) of family labor spent on livestock activity | 848 | 4.05 | 1.60 | 0.16 | 7.98 |
| *Log* of the amount (days) of hired labor used in livestock activity | 848 | 0.11 | 0.67 | 0.00 | 6.29 |
| Income transfers to farmers from PROGRAN (thousands of MX$) | 848 | 0.15 | 1.52 | 0.00 | 32.00 |
| **Corn production** |  |  |  |  |  |
| Native seed use (1=Yes, 0=No) | 328 | 0.82 | 0.39 | 0.00 | 1.00 |
| Credit access to cultivate (1=Yes, 0=No) | 328 | 0.06 | 0.24 | 0.00 | 1.00 |
| Use of animal traction (1=Yes, 0=No) | 328 | 0.32 | 0.47 | 0.00 | 1.00 |
| Access to irrigation (1=Yes, 0=No) | 328 | 0.26 | 0.44 | 0.00 | 1.00 |
| *Log* of cropped area | 328 | 0.34 | 1.06 | -2.66 | 3.26 |
| *Log* of the amount (days) of family labor spent to cultivate | 328 | 3.44 | 1.22 | 0.00 | 6.61 |
| *Log* of the amount (days) of hired labor used to cultivate | 328 | 1.05 | 1.33 | 0.00 | 4.80 |
| Machinery used during the harvest | 328 | 0.13 | 0.33 | 0.00 | 1.00 |
| Expenditure in fertilizers, manure and other agro-chemicals (thousands of MX$) | 328 | 2.37 | 4.91 | 0.00 | 31.48 |
| Machinery used before the harvest (different from animal traction) | 328 | 0.48 | 0.50 | 0.00 | 1.00 |
