# Supplementary material for: Estimating shadow prices in economies with multiple market failures
Source: PLoS One. 2023 Nov 6;18(11):e0293931. doi: 10.1371/journal.pone.0293931 (PMC10627447; doi:10.1371/journal.pone.0293931)
Supplement: S1 Appendix — (DOCX) [file pone.0293931.s002.docx]

**S1 Appendix. Solution to the Theoretical Model**

The optimization problem faced by the producer-consumer household described in section 2 is to choose the household’s consumption choices ($C_{x}, C_{s}$), production decisions ($H_{s}, H_{c}$), and time allocation ($M,F_{s},F_{c}$) that:

| $Max U\left[ C_{x};C_{s};T-M-F_{s}-F_{c};B \right]$ | (S1.1) |
| --- | --- |

Subject to:

| $p_{x}C_{x}-p_{c}Q_{c}\left( F_{c},H_{c},K_{c} \right)+wH_{c}-w^{m}M-p_{s}\left( Q_{s}\left( F_{s},H_{s},K_{s} \right)-C_{s} \right)+wH_{s}=V$ | (S1.2) |
| --- | --- |
| $C_{s}{-Q}_{s}\left( F_{s},H_{s},K_{s} \right)\leq0$ | (S1.3) |
| $M\leq\delta$ | (S1.4) |

Where:

| $T=L+M+F_{s}+F_{c}$ | (S1.5) |
| --- | --- |

Applying the Kuhn-Tucker conditions [1], there exist multipliers $\lambda, \mu_{s}, and \mu$ associated with the equations (S1.2), (S1.3), and (S1.4) respectively, such that:

| $\frac{\partial U}{\partial C_{x}}\boldsymbol{=} \lambda p_{X}$ | (S1.6) |
| --- | --- |
| $\frac{\partial U}{\partial M}=-\lambda w^{m}+\mu$ | (S1.7) |
| $\frac{\partial U}{\partial F_{c}}\boldsymbol{=} -\lambda p_{c}\frac{\partial Q_{c}\left( F_{c},H_{c},K_{c} \right)}{\partial F_{c}}$ | (S1.8) |
| $\frac{\partial U}{\partial H_{c}}\boldsymbol{=} \lambda\left( -p_{c}\frac{\partial Q_{c}\left( F_{c},H_{c},K_{c} \right)}{\partial H_{c}}+w \right)$ | (S1.9) |
| $\frac{\partial U}{{\partial C}_{s}}\boldsymbol{=} \lambda p_{s}+\mu_{s}$ | (S1.10) |
| $\frac{\partial U}{\partial F_{s}}\boldsymbol{=} -\left( \lambda p_{s}+\mu_{s} \right)\frac{{\partial Q}_{s}\left( F_{s},H_{s},K_{s} \right)}{\partial F_{s}}$ | (S1.11) |
| $\frac{\partial U}{\partial H_{s}}\boldsymbol{=} \lambda w-{(\lambda p}_{s}+\mu_{s})\frac{{\partial Q}_{s}\left( F_{s},H_{s},K_{s} \right)}{\partial H_{s}}$ | (S1.12) |

From equation (S1.6), we deduce:

| $\lambda=\frac{1}{p_{X}}\frac{\partial U}{\partial C_{x}}$ | (S1.13) |
| --- | --- |

Furthermore, using the relation:

| $\frac{\partial U}{\partial M}=\frac{\partial U}{\partial L}\frac{\partial L}{\partial M}=-\frac{\partial U}{\partial L}$ | (S1.14) |
| --- | --- |

Together with (S1.6) and (S1.7), we obtain:

| ${\frac{\partial U}{\partial L}}/{\frac{\partial U}{\partial C_{x}}}\boldsymbol{=}\frac{1}{p_{x}}\left( w^{m}-\frac{\mu}{\lambda} \right)$ | (S1.15) |
| --- | --- |

Consumer theory establishes that, at the optimum, the relationship between the marginal utilities of two goods is equal to the proportion of their prices [2,3]. Thus, from (S1.15), we deduce that $w^{m}-\frac{\mu}{\lambda}$​ must be the (shadow) price of leisure *L*, denoted $w^{*}$, which by definition is the shadow wage of family labor. Thus:

| $w^{*}=w^{m}-\frac{\mu}{\lambda}$ | (S1.16) |
| --- | --- |

Using (S1.7), (S1.14) and considering that $\frac{\partial U}{\partial F_{c}}\boldsymbol{=}\frac{\partial U}{\partial L}\frac{\partial L}{{\partial F}_{c}}\boldsymbol{=-}\frac{\partial U}{\partial L}$, equation (S1.8) simplifies to:

| $-\lambda w^{m}+\mu\boldsymbol{=} -\lambda p_{c}\frac{\partial Q_{c}\left( F_{c},H_{c},K_{c} \right)}{\partial F_{c}}$ | (S1.17) |
| --- | --- |

From (S1.16), we infer:

| $w^{*}=p_{c}\frac{\partial Q_{c}\left( F_{c},H_{c},K_{c} \right)}{\partial F_{c}}$ | (S1.18) |
| --- | --- |

Given that $\frac{\partial U}{\partial H_{c}}\boldsymbol{=}0$, from (S1.9) we have:

| $w=p_{c}\frac{\partial Q_{c}\left( F_{c},H_{c},K_{c} \right)}{\partial H_{c}}$ | (S1.19) |
| --- | --- |

Equations (S1.10) and (S1.6) imply:

| ${\frac{\partial U}{{\partial C}_{s}}}/{\frac{\partial U}{\partial C_{x}}}\boldsymbol{=}\left( p_{s}+\frac{\mu_{s}}{\lambda} \right)/{p_{X}}$ | (S1.19) |
| --- | --- |

Using reasoning similar to the shadow wage, from the above equation, we deduce that the shadow price of good *s* consumed by the household is $p_{s}+\frac{\mu_{s}}{\lambda}$, denoted $p_{s}^{*}$​. Thus:

| $p_{s}^{*}\boldsymbol{=}p_{s}+\frac{\mu_{s}}{\lambda}$ | (S1.20) |
| --- | --- |

The above, together with (S1.7), (S1.11), (S1.14), (S1.16) and the relation $\frac{\partial U}{\partial F_{s}}\boldsymbol{=}\frac{\partial U}{\partial L}\frac{\partial L}{{\partial F}_{s}}\boldsymbol{=-}\frac{\partial U}{\partial L}$​, leads to:

| $p_{s}^{*}=\frac{w^{*}}{\frac{\partial Q_{s}\left( F_{c},H_{c},K_{c} \right)}{\partial F_{s}}}$ | (S1.21) |
| --- | --- |

Additionally, given that $\frac{\partial U}{\partial H_{s}}\boldsymbol{=}0$, from (S1.12) and (S1.20) we obtain:

| $w=p_{s}^{*}\frac{{\partial Q}_{s}\left( F_{s},H_{s},K_{s} \right)}{\partial H_{s}}$ | (S1.22) |
| --- | --- |

Finally, it should be mentioned that the complementary slackness conditions add the following restrictions:

| $\mu_{s}\left( C_{s}{-Q}_{s}\left( F_{s},H_{s},K_{s} \right) \right)=0$ | (S1.23) |
| --- | --- |
| $\mu\left( M-\delta\right)=0$ | (S1.24) |

**References**

1. Mas-Colell, A, Whinston, M D, Green, J R. Microeconomic theory. New York: Oxford university press; 1995.
2. Jacoby HG. Shadow wages and peasant family labour supply: an econometric application to the Peruvian Sierra. The Review of Economic Studies. 1993;60:903-21. doi: <https://doi.org/10.2307/2298105>.
3. Varian, H R. Microeconomía intermedia: un enfoque actual. Alpha Editorial; 2016.
